# Profile of children referred to primary health care physiotherapy: a longitudinal observational study in Norway

**DOI:** 10.1186/s12913-020-05988-8

**Published:** 2021-01-06

**Authors:** Kari Anne I. Evensen, Siw Sellæg, Anne-Cath Stræte, Anne E. Hansen, Ingebrigt Meisingset

**Affiliations:** 1 Department of Public Health and Nursing, NTNU, Trondheim, Norway; 2 Department of Clinical and Molecular Medicine, NTNU, Trondheim, Norway; 3 Unit for Physiotherapy Services, Trondheim Municipality, Trondheim, Norway; 4 Department of Physiotherapy, Oslo Metropolitan University, Oslo, Norway

**Keywords:** Children, Cohort, Database, Electronic registration, Goal attainment, Municipality, Physiotherapy, Child health care centre, Public health nurse, Primary health care services

## Abstract

**Background:**

Physiotherapy services are an important part of the primary health care services for children, serving a broad spectrum of children referred from different sources and for a variety of reasons. There is limited knowledge about their characteristics and outcome. The aim of this study was to describe the profile, i.e. referral patterns, baseline demographical and clinical characteristics, as well as treatment outcome at follow-up 6 months after baseline, of children receiving physiotherapy in primary health care.

**Methods:**

Children referred to primary health care physiotherapy in a large municipality in Norway were invited to participate in this longitudinal observational study. The children’s demographics, referral sources, causes of referral, functional diagnoses, influence on their daily activities, main goals and planned treatments were registered at baseline. Goal attainment and treatment compliance were registered at follow-up maximum 6 months after baseline.

**Results:**

The physiotherapists registered baseline characteristics for 148 children. Parent-reported data at baseline were available for 101 (68.2%) of these children. Children were mainly referred from child health care centres (*n* = 74; 50.0%), hospital (*n* = 25; 16.9%) and kindergarten (*n* = 22; 14.9%). The most frequent causes of referral were concerns for motor development (*n* = 50; 33.8%), asymmetry (*n* = 40; 27.0%) and orthopaedic conditions (*n* = 25; 16.9%). Eighty-one (54.7%) children were below the age of 1 year. There was partly agreement between causes of referral and the physiotherapists’ functional diagnoses. Parents of 69 (71.1%) children reported that their child’s daily activities were little to not at all affected by the problem or complaint for which they were referred. Follow-up data were registered for 64 children. The main treatment goal was achieved in 37 (57.8%) and partly achieved in 26 (40.6%) children and the treatment was carried out as planned in 55 (87.3%) children.

**Conclusions:**

The large variation in the profile of children receiving physiotherapy in a primary health care setting in Norway shows how primary health care physiotherapists can contribute to fulfil the broad purpose of the primary health care services.

**Trial registration:**

ClinicalTrials.gov Identifier: NCT03626389. Registered on August 13th 2018 (retrospectively registered).

**Supplementary Information:**

The online version contains supplementary material available at 10.1186/s12913-020-05988-8.

## Background

Physiotherapy services are an important part of the primary health care services for children, ranging from health promotion to prevention, treatment and rehabilitation. As primary health care addresses whole-person’s health needs, and not just specific diseases [1], physiotherapists (PTs) serve a broad spectrum of children and their families. Further, primary health care services are offered through comprehensive and coordinative care carried out in people’s everyday environment [1]. Thus, most industrialised countries in Europe provide low threshold free of charge child health care interventions involving the parents, and often a multidisciplinary team of health professionals [2–4].

In Norway, all municipalities should provide universal health services in child health care centres, school health services and youth health centres [4, 5], and the physiotherapy services are partly carried out at the child health care centres, at home, in kindergarten or at school. The primary health care PTs in Norway have a close collaboration with, and can receive referrals from, other professionals. As in many other countries in Europe [3, 6], the public health nurses have a key role as they are the primary care providers who meet with the families first and most frequently [7, 8]. Children may also be referred from general practitioners, occupational therapists, health professionals in specialised health care services or personnel in kindergarten or school. Physiotherapy may also be initiated on basis of parental concern, as eliciting and attending to parental concerns is a key element of effective developmental surveillance and in line with international best practice [7].

Children are referred to primary health care PTs for a variety of reasons. Suspected motor delay or motor problems should be timely referred [8], as there is an assumption that early intervention enhancing brain plasticity may be particularly beneficial [9–12]. Also, motor difficulties may have consequences in other domains beyond motor skills [13, 14]. Other causes of referral to physiotherapy may range from asymmetry, including positional preferences, plagiocephaly or congenital muscular torticollis, orthopaedic conditions, including concerns for foot alignment, and prevention of obesity to chronic or neurological conditions and need for habilitation services. However, not all causes of referral may affect the child’s daily life and the PT may evaluate the child as typically developing and not in need for physiotherapy. Thus, the goal and plan for treatment for children referred to primary health care PTs can vary substantially. As much of the existing literature on physiotherapy for children concerns descriptions and intervention for specific diagnoses, often carried out in specialised health care, there is limited knowledge of characteristics and treatment outcome of the broad spectrum of children receiving primary health care physiotherapy. Such knowledge can be used to evaluate referral patterns in order to improve the coordinative care of children in primary health care services.

The primary aim of this study was to describe the profile, i.e. referral patterns, baseline demographic and clinical characteristics, of children receiving physiotherapy in primary health care. Secondly, we examined the influence of the problem or complaint on the child’s daily activities and the PT’s functional diagnosis. Thirdly, we assessed goal setting and plan for treatment as well as goal attainment and treatment compliance at follow-up 6 months after baseline for children where physiotherapy was initiated.

## Methods

### Design and setting

Through the Research program for Physiotherapy in Primary Health Care, the FYSIOPRIM, a set of standardised methods and tools have been developed, enabling studies of clinical courses for patients receiving primary care physiotherapy [15]. The present study is a longitudinal observational study of children referred to physiotherapy in Trondheim Municipality, which is in the middle part of Norway. Trondheim currently has around 205,000 inhabitants and is the third largest municipality in Norway.

Baseline data were prospectively collected for newly referred children during a period of 12 consecutive months from May 1^st^ 2016 through April 30^th^ 2017 with follow-up data collected maximum 6 months after baseline. The study was conducted according to the Helsinki Declaration. Written informed consent was obtained from one or both parents of all children. Ethical approval was granted by the Regional committees for Medical and Health Research Ethics in Norway (REC no. 2013/2030).

### Participants

**Fig. 1:**
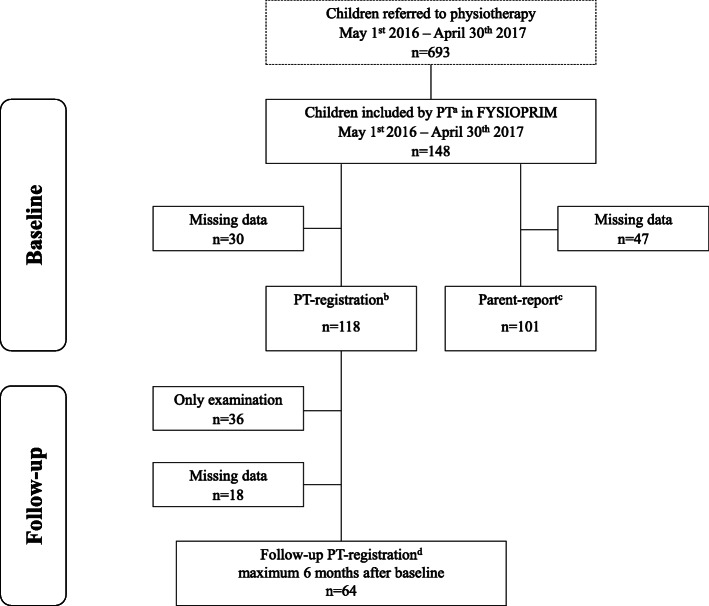
Flow chart of children included in FYSIOPRIM. FYSIOPRIM = Research program for Physiotherapy in Primary Health Care. PT = Physiotherapis. ^a^Initial PT-registration included age, sex, referral source and cause of referral. ^b^Baseline PT-registration at the first consultation included PT’s functional diagnosis, whether the child was starting physiotherapy or had an examination only, and main treatment goal and planned treatment identified by the PT and parents together. ^c^Baseline parent-report after the first consultation included the child’s living situation and daily arena, child and parents’ country of birth, parents’ highest level of education, whether the child was born preterm, use of hospital services, pain and influence on daily activities. ^d^Follow-up PT-registration included number of consultations, goal attainment and treatment compliance assessed by the PT and parents together, and whether the child continued physiotherapy

All children aged 0–18 years referred to physiotherapy in Trondheim Municipality, Norway, were eligible for inclusion. A total of 693 children was referred to physiotherapy during the 12-month inclusion period. Exclusion criteria were parents not able to understand Norwegian or English as the consent information for the project was only available in these languages, and the parent-report questionnaires were only available in Norwegian. Flow of children included in FYSIOPRIM is shown in Fig. 1.

### Data collection procedure

The parents of all eligible children were asked to participate prior to or during the first consultation with the PT. A tablet application was used for electronic data collection, and parents also had the opportunity to answer questionnaires through an e-mail link [15]. At baseline, we used data registered by the PT at the first consultation and parent-reported data registered after the first consultation. At follow-up, we only used data registered by the PT, due to a substantial amount of missing parent-reported data at follow-up. Details of questions from FYSIOPRIM used in this study are shown in Additional File 1.

### Baseline assessment

At baseline, the PT registered the child’ sex, age, referral source and cause of referral. Categories of referral source and cause of referral were predefined by the physiotherapy services (Additional File 1). In this study, referral source was categorised into “child health care centre”, “hospital”, “kindergarten”, “school”, “school health care services”, “general practitioner”, “proxy/parent” and “other”. Cause of referral was categorised into “motor development”, “asymmetry”, “orthopaedics”, including gait and foot alignment, “established neurological diagnosis/syndrome”, “advice in physical activity” and “other”, including heart and lung disease, overweight, juvenile arthritis, cancer, fractures, pain, myalgic encephalomyelitis, referral for assistive devices, multidisciplinary assessment or other.

The PT’s functional diagnosis was registered as free text. The PT and the parents together identified the main goal and plan for treatment, which were registered as free text. Specific for this study, we reviewed all answers and defined categories that reflected the variation in the PT’s functional diagnosis, treatment goal and treatment (Table 1). The PT’s functional diagnosis was categorised into “normal findings”, “motor difficulties”, “asymmetry” (including positional preferences, plagiocephaly or congenital muscular torticollis), “foot alignment”, “established diagnosis/syndrome” and “other”. We categorised the main goal into “no further follow-up”, “normalise or optimise motor development”, “achieve symmetrical movements”, “pain reduction”, “further examinations” and “other”. Plan for treatment was categorised into “no need for treatment”, “further examinations”, “advice/guidance of parents”, “stimulation to active movements”, “adaptation of the environment and positional support”, “stretching” and “other”.

**Table 1:** Examples of categorisation of the physiotherapist’s (PT’s) functional diagnosis, main goal for treatment and treatment plan registered as free text

Categorisation	Free text
**PT’s functional diagnosis**	
Normal findings	No diagnosis; Not relevant; Normal development; None; Normal joint conditions
Motor difficulties	Bodily unrest and attention deficit; Struggles with gross motor skills; Immature movement pattern; Slightly delayed motor development according to age; Delayed fine motor development; Delayed gross motor development; Struggles in prone position; Reduced head control
Asymmetry	Infant asymmetry; Positional infant asymmetry; Neck asymmetry; Torticollis; Favorite side; Asymmetrical movement development; Lateral flexion to the right side; Asymmetry
Foot alignment	Stiff ankles, toe-walking; Intoeing; Asymmetrical running pattern and stiff ankle; Increased valgus in foot; Flexible flatfoot
Established diagnosis/syndrome	Down's syndrome; Rare diseases (not shown due to anonymity)
Other	Birth asphyxia; Pain problem; Tension headache; Stiff neck; Ankle fracture; Diffuse leg pain
**Main goal for treatment**	
No further follow-up	Only examination; No need for follow-up; Case closed
Normalise or optimise motor development	Age-adequate motor development; Independent walking; Normal motor development; Improve independence in fine- and gross motor tasks; Improve writing; Normalise running pattern; Impact walking and running
Achieve symmetrical movements	Symmetrical motor development; Symmetrical neck position; Symmetry in neck; Equal range of motion to both sides; Full range of motion bilaterally; Become equally strong in active lateral flexion to both sides; Become symmetrical in prone position; Achieve symmetry of head movements
Pain reduction	Reduce pain in neck and back; Become pain free, become aware of tension; Reduce pain and fatigue
Further examinations	Evaluate need for follow-up; Evaluation of fine motor function as part of further assessment; Mapping of motor skills; Assessment of feet; Assessment/evaluation of fine motor function and coordination; Determine need for further follow-up/assessment; Examine the cause of intoeing and determine need for follow-up; Find the cause of asymmetry and stiffness in ankle; Examination of neck; Examination and evaluation; Consider further follow-up by PT;
Other	Maintain function; Participation in suitable and pleasurable leisure activities; Improve sleep and school day functioning; Improve range of motion in dorsiflexion of the ankle; Prevent problems due to week muscles; Avoid stiffening of shoulder; Monitor development
**Treatment plan**	
No need for treatment	No plan for treatment; No goal for treatment; No further follow-up
Further examinations	Assessment; Examination; Observation and assessments; Mapping of causes; Observe in kindergarten; Observe in school; Evaluate motor development
Advice/guidance of parents	Guidance of mother; Guidance of parents; Guidance kindergarten/home; Talk to child, mother and school; Ensure that parents are given knowledge-based information about motor development
Stimulation to active movements	Stimulate both sides in daily activities; Stimulation on the child’s left side; Stimulate to symmetrical head control and varied positions; Stimulation; Stimulate to active rotation of the head to the left; Active lateral flection to the right in various position; Functional movements
Adaptation of the environment and positional support	Adaptation in school; Adaptation; Supine position with adequate support to promote head in midline; Facilitate prone position; Facilitate varied motor development; Support for supine position
Stretching	Stretching
Other	Normal activity level; Balance, strength and stability; Variation in position; Understand the condition; Referral to occupational therapist

The parents reported on the child’s living situation (living with one/both parent(s), siblings), the child’s daily arena (home/kindergarten/school), child and parents' country of birth, parents’ highest level of education (primary school or lower/high school/up to 4 years of college or university/more than 4 years of college or university), whether the child was born preterm (< 37 weeks of gestation, yes/no), and use of hospital services the last 12 months (yes/no). Pain was assessed with the question: “If applicable, does the child have pain?”, with yes/no as response options, and a numerical rating scale (0–10, where 0 indicated no pain and 10 worst possible pain) if yes. The influence on daily activities was assessed by the question: “How much does the problem or complaint affect the child’s daily activities?”, with response options on a 6-point Likert scale (very much/much/some/little/very little/not at all).

### Follow-up assessment

At follow-up, the PT and the parents together assessed goal attainment on a 3-point Likert scale by the question: “To which extent was the main treatment goal achieved?” The response options were 1) achieved, 2) partly achieved and 3) not achieved. Treatment compliance was assessed by the question: “To which extent was the treatment carried out as planned?” with the response options 1) performed, 2) partly performed and 3) not performed.

### Statistical analyses

Data were analysed in SPSS version 25 (IBM SPSS Statistics. Statistical Package for Social Sciences) and STATA 15.1 (StataCorp. 2017. Stata Statistical Software: Release 15. College Station, TC: StataCorp LLC). We used descriptive statistics, i.e. numbers and proportions, n (%), to describe the baseline demographical and clinical characteristics of the children. Further, we used cross tabulations and proportions to describe the relationships between the following variables; i) referral source with age and cause of referral; ii) cause of referral with influence on daily activities, the PT’s functional diagnosis and whether the child had an examination only; and iii) the PT’s functional diagnosis with the main goal, planned treatment and goal attainment. To assess if children with missing data at follow-up were different from those with complete data, we compared sex, age, referral source and cause of referral as well as living situation, daily arena, child and parents' country of birth, parents' highest level of education, whether the child was born preterm, use of hospital services, pain and influence on daily activities at baseline by using t-test for continuous data and chi-square test for categorical data. A significance level of 0.05 was used.

## Results

### Demographical and clinical characteristics

Table 2 shows the demographical characteristics of the children included in FYSIOPRIM and Table 3 gives an overview of causes of referral across referral sources. At baseline, 148 newly referred children had their demographic information registered (Fig. 1). Approximately half were males and below the age 1 year (Table 2).

**Table 2:** Demographical characteristics of children included in FYSIOPRIM

Baseline PT-registration^a^ (*n* = 148)	n	(%)
Male sex	85	(57.4)
Age		
0–11 months	81	(54.7)
1–2 years	25	(16.9)
3–5 years	19	(12.8)
6–8 years	8	(5.4)
9–11 years	10	(6.8)
12–16 years	5	(3.4)
Baseline parent-report^b^ (*n* = 101)	n	**(%)**
Living with both parents	91	(90.1)
Siblings	63	(62.4)
Child’s daily arena		
At home	62	(61.4)
Kindergarten	25	(24.8)
School	14	(13.9)
Born in Norway	99	(98.0)
Mother born in Norway^c^	86	(86.0)
Father born in Norway^d^	85	(85.0)
Mother with higher education^e^	75	(75.0)
Father with higher education^f^	67	(67.7)
Preterm born (before week 37)	22	(21.8)
Hospital services last 12 months^g^	25	(25.5)
Pain^h^	15	(15.2)

*FYSIOPRIM* Research program for Physiotherapy in Primary Health Care; *PT* Physiotherapist

^a^Registered by the physiotherapist at the first consultation

^b^Reported by the parents after the first consultation

^c^Missing data for one mother

^d^Missing data for one father

^e^Missing data for one child

^f^Missing data for two children

^g^Missing data for three children

^h^Missing data for two children, only reported proportion due to few children reporting pain

**Table 3:** Overview of causes of referral across referral sources for children included in FYSIOPRIM

Cause of referral	Motor development		Asymmetry^a^		Orthopaedics		Preterm		Diagnosis^b^/syndrome		Advice PA		Other^c^		Total	
Referral source	n	(%)	n	(%)	n	(%)	n	(%)	n	(%)	n	(%)	n	(%)	n	(%)
Child health care centre	16	(32.0)	36	(90.0)	13	(52.0)	6	(66.7)	–	–	–	–	3	(20.0)	74	(50.0)
Hospital	6	(12.0)	3	(7.5)	1	(4.0)	3	(33.3)	4	(80.0)	2	(50.0)	6	(40.0)	25	(16.9)
Kindergarten	17	(34.0)	–	–	3	(12.0)	–	–			–	–	2	(13.3)	22	(14.9)
School	5	(10.0)	–	–	1	(4.0)	–	–	1	(20.0)	–	–	1	(6.7)	8	(5.4)
School health care services	2	(4.0)	–	–	4	(16.0)	–	–	–	–	1	(25.0)	1	(6.7)	8	(5.4)
General practitioner	1	(2.0)	–	–	2	(8.0)	–	–	–	–	–	–			3	(2.0)
Proxy/parent	2	(4.0)	1	(2.5)	1	(4.0)	–	–	–	–	–	–	1	(6.7)	5	(3.4)
Other	1	(2.0)			–	–	–	–	–	–	1	(25.0)	1	(6.7)	1	(0.6)
Total	50	(33.8)	40	(27.0)	25	(16.9)	9	(6.1)	5	(3.4)	4	(2.7)	15	(10.1)	148	(100)

*FYSIOPRIMM* Research program for Physiotherapy in Primary Health Care; *PA* Physical activity

^a^Positional preference of head or truncus, congenital muscular torticollis

^b^Established neurological diagnosis

^c^Heart and lung disease, overweight, juvenile arthritis, cancer, fractures, pain, myalgic encephalomyelitis, referral for assistive devices, multidisciplinary assessment or other

Of the 81 children below 1 year of age, 58 (71.6%) were referred from child health care centres and 16 (19.8%) from the hospital, while the rest was referred from parents, kindergarten and general practitioner (data not shown). The main cause of referral was concern for motor development in one third of the children (33.8%), mainly referred from the child’s kindergarten or child health care centre (Table 3), followed by asymmetry in 40 (27.0%) children, and orthopaedic conditions in 25 (16.9%) children. The latter two groups of children were mainly referred from child health care centres (Table 3).

More than 90% of the children were living with both parents, and more than 60% had siblings (Table 2). Reflected by the age, most children had their home as their daily arena, one fourth were in kindergarten and one sixth in school. One fifth of the children were born preterm, two thirds of these were referred from child health care centres and one third from the hospital (Table 3). A quarter of the children had received hospital services the last 12 months and 15 (15.2%) children had pain. All but two  children and most parents were born in Norway.

### Influence on daily activities and functional diagnosis

**Fig. 2:**
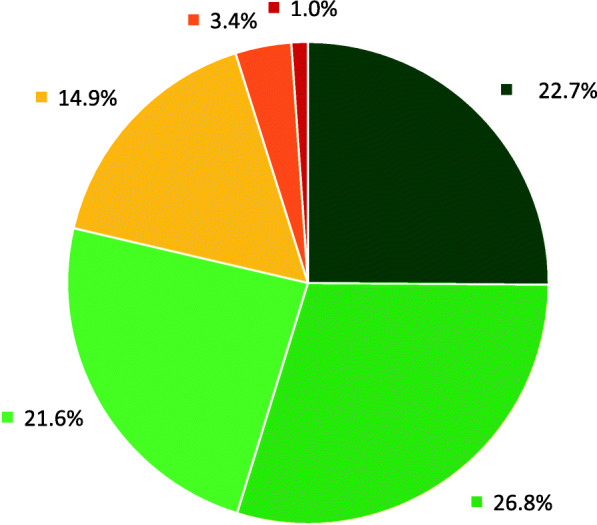
Parent-reported influence of the problem or complaint on the child’s daily activities (*n* = 97)

Parents of 69 (71.1%) children reported that the problem or complaint, for which the child had been referred to physiotherapy, affected the child’s daily activities little, very little or not at all (Fig. 2). The child’s daily activities were some, much or very much affected only in one of eight (12.5%) children referred for motor development concerns, one sixth (17.4%) of the children referred for asymmetry and half (50%) of the children referred for orthopaedic conditions (data not shown).

The PT’s functional diagnosis was registered for 108 (73.0%) of the included children. Normal findings were registered in 24 (22.2%) of them. In 26 (24.1%) children, the functional diagnosis was motor difficulties, in 30 (27.8%) asymmetry and in nine (8.3%) children foot alignment. Ten (9.3%) children had established diagnoses/syndromes and nine (8.3%) had other functional diagnoses. Of the 36 children referred for motor development concerns, 23 (63.9%) were classified as having motor difficulties by the PT, while eight (22.2%) had normal findings, three (8.3%) had a functional diagnosis of asymmetry or foot alignment and two (5.6%) had other functional diagnoses (data not shown). Of the 27 children referred for asymmetry, 24 (88.9%) were classified as having asymmetry by the PT, while the rest (11.1%) had normal findings. Of the 20 children referred for orthopaedic concerns, seven (35.0%) had a functional diagnosis of foot alignment, while 11 (55.0%) were classified as having normal findings by the PT and two (10.0%) had other functional diagnoses (data not shown).

### Goal setting, plan for treatment and goal attainment at follow-up

Of the 118 children with PT-registered data (Fig. 1), only examination was registered for 36 (30.5%). Of these, 18 (50.0%) were referred for orthopaedic conditions, constituting 72% (18 of 25) of all children referred for orthopaedic conditions, ten (27.8%) for motor development concerns, six (16.7%) for asymmetry and two (5.5%) for other reasons. Main treatment goal was registered for 109 children, whereof 16 (14.7%) children were among those with only examination and the reported goal was no further follow-up. The most frequent goal was to normalise or optimise motor development in 34 (31.2%) children, followed by achieving symmetrical movements in 31 (28.4%). Further examinations were registered as the main goal for 14 (12.8%) children. For four children (3.7%) the goal was pain reduction and for ten (9.2%) children other.

Plan for treatment was registered for 106 children. Six (5.7%) of these were among those with only examination and did not need any treatment. Further examinations were registered as the planned treatment for 17 (16.0%) children. For 33 (31.1%) children, the plan for treatment involved advice/guidance of parents, and stimulation to active movements in 25 (23.6%) children. Further, adaptation of the environment and positional support were registered for 11 children (10.4%), stretching for two children (1.9%) and other for 12 (11.3%) children.

Of the 25 children whose functional diagnosis was motor difficulties, the main goal was to normalise or optimise motor development in the majority (*n* = 20, 80.0%) and the planned treatment was advice/guidance of parents in 15 (60.0%) and adaptation of the environment in another four (16.0%). Of the 30 children with functional diagnosis of asymmetry, the main goal was to achieve symmetrical movements in 23 (76.7%), and the planned treatment involved stimulation to active movements in 18 (60.0%) children, followed by advice/guidance in nine (30.0%) and adaptation of the environment in two (6.7%). Of the 8 children with foot alignment as functional diagnosis, the main goal varied from no need for follow-up in two (25.0%) children, further examinations in three (37.5%) children and guidance of parents, pain reduction or other for the rest, and the planned treatment varied accordingly (data not shown).

Table 4 shows the follow-up data of the included children. Of the 64 children registered at follow-up, seven (13.2%) had only one consultation. Around a quarter had 2–3 consultations and 4–6 consultations, respectively. A third of the children continued physiotherapy after follow-up registration at 6 months (Table 4). Of these, four (22.2%) children were initially referred for motor development concerns, four (22.2%) children for neurological conditions including established diagnoses, four (22.2%) children for asymmetry, two (11.1%) for prematurity, one (5.6%) for orthopaedic condition and 3 (16.7%) children for other reasons.

**Table 4:** Follow-up data of children included in FYSIOPRIM

Follow-up PT-registration (*n* = 64)	n	(%)
Physiotherapy consultations^a^		
1	7	(13.2)
2–3	15	(28.3)
4–6	15	(28.3)
7–9	8	(15.1)
> 9	8	(15.1)
Continuing physiotherapy at 6 months^a^	18	(34.0)
Main treatment goal^b^		
Achieved	37	(57.8)
Partly achieved	26	(40.6)
Not achieved	1	(1.6)
Treatment compliance^b,c^		
Performed	55	(87.3)
Partly performed	5	(7.9)
Not performed	3	(4.8)

*FYSIOPRIM* Research program for Physiotherapy in Primary Health Care; *PT* Physiotherapist

^a^Missing data for 11 children

^b^Assessed by the physiotherapist and parents together

^c^Missing data for one child

The main treatment goal was achieved in 37 (57.8%) and partly achieved in 26 (40.6%) children (Table 4). Of the children with follow-up data whose PT’s functional diagnoses were motor difficulties (*n* = 20) and asymmetry (*n* = 22), 12 (60.0%) and 16 (72.7%) children, respectively, achieved the goal, while the rest partly achieved the goal. In nearly 90% of the children the treatment was carried out as planned (Table 4).

### Missing data

There were no significant baseline differences between children with and without follow-up data regarding sex, referral source, living with both parents, having siblings, child and parents' country of birth, parents' highest level of education, pain, or influence on daily activities. Children without follow-up data were older (*p* = 0.024) and in school (*p* = 0.05), a higher number was referred for orthopaedic conditions (*p* < 0.001), born at term (*p* = 0.003) and had not received hospital services the last 12 months (*p* = 0.037).

## Discussion

The present study is the first to describe a broad spectrum of children referred to physiotherapy during 1 year in a primary health care setting in Norway. As expected, the children were heterogeneous in terms of age and cause of referral. Most were referred from child health care centres for concerns regarding motor development, asymmetry or orthopaedic conditions (including concerns for foot alignment), even though most parents reported that the problem for which their child was referred had little or no influence on their daily activities. The cause of referral and the PT’s functional diagnosis overlapped to a great extent when it came to motor development and asymmetry, but more than half of the children referred for orthopaedic conditions were classified as having normal findings by the PT. By far the majority achieved or partly achieved their main treatment goal and the treatment was carried out as planned.

The high proportion of children referred from child health care centres reflects the key role of the public health nurses in Norway that see the families and children routinely during the first years of life [4]. However, most parents reported that their child had no pain and was little affected by the problem, and about a third of the children only had an examination or one consultation, which may question the rationale for referral. On the other hand, this may be inherent in the cause of referral as most infants with asymmetry are not in pain and given the children’s young age, the problem may not (yet) be affecting their daily life. Furthermore, an examination of the child and reassurance of parents could still play an important role and prevent other attempts to get help or reassurance from other health care providers. It also fits well with the goal that the primary health care services should provide low threshold services[4].

In our study, there was a great overlap between causes of referral and the PT’s functional diagnoses regarding concerns for motor development and asymmetry, indicating that the nurses and the PTs judged these conditions similarly. This is likely to be a result of a well-functioning interdisciplinary collaboration. In Sweden, public health nurses have worked systematically to implement a screening tool for motor development in collaboration with PTs, which has shown to identify children in need for PT [16]. In Norway, there has been a shift towards more group consultations and less individual appointments in the public child health care programme [4]. Group consultations has shown to provide a setting for building trust, quality of relationships and collaboration between professionals, factors which are identified as key characteristics of knowledge transfer and exchange in health care [17, 18]. At joint group consultations by PTs and public health nurses at 4 months of age in Trondheim Municipality, the PTs have addressed typical motor development by emphasising that every child is developing at their own pace and by illustrating how development may be affected by several factors within the child as well as the interplay between the child, the activities that the child does and environmental factors [19]. This may have led to a common understanding of typical motor development among PTs and public health nurses.

In contrast, more than half of the children referred regarding concerns for foot alignment were classified as having normal findings by the PT, and the majority of these needed only an examination. One explanation may be that these cases are more difficult to evaluate for the nurses and they may lack sufficient knowledge. For most foot alignment cases there is no conservative treatment to offer, but simply to observe over time and expect the condition to resolve spontaneously as the child grows older [20–22]. In order to implement knowledge regarding examination and treatment for the most common causes of referral from child health care centers; i.e. infant asymmetry, intoing, flatfoot and toe-walking, the Unit for Physiotherapy Services in Trondheim Municipality has during the recent years developed and adapted clinical guidelines to local conditions [20–24]. The guidelines were communicated to the public health nurses, but they were not involved in the development of the guidelines. The findings from Sweden underline the importance of including other relevant health care professional when implementing new measures in a clinical setting [25]. This could increase compliance to clinical guidelines, improve collaboration and result in more timely referrals between health care professionals in child health care services.

Of the children where physiotherapy was initiated, about 70% had 6 or less consultations during the six-month period. This supports the PT’s role in child health care services as low threshold services serving young children where the potential for early intervention is high and that the treatment goals are achieved despite using relatively few resources. Further, the large variation in the registered functional diagnoses, main treatment goals and planned treatment shows the heterogeneity of the children receiving primary health care physiotherapy. It is reassuring that the main treatment goal and planned treatment for asymmetry and foot alignment were in accordance with our clinical guidelines [20–23], which are based on international literature [26–29]. In Trondheim Municipality, the principles for physiotherapy for children is based on a family-centred approach [30]. The relatively low frequency may therefore be consistent with follow-up being an integrated part of the child’s daily activities at home, kindergarten or school, where the PT’s role is to guide parents and other caregivers on how to implement the treatment plan in daily life. About 40% of the children needed continued physiotherapy services after the six-month follow-up, and numbers indicated that at least half of this children may be children with more complex problems, such as sustained motor development problems, established diagnoses/syndromes and preterm born children. The latter group may be followed by a PT for surveillance even though they may not have current problems [31]. The large variation in characteristics of referred children, goal setting, treatment plan and frequency of physiotherapy shows how primary health care PTs can contribute to fulfil the broad purpose of the primary health care services [1].

Strengths of the present study were the systematic data collection of children receiving primary health care physiotherapy services, information about referral patterns, inclusion of parent-reported data, and follow-up registration of goal attainment and treatment compliance. Several of the questions included in the current study are not validated for use among parents and their children. We planned to include validated and widely used questionnaires and tools, such as the Alberta Infant Motor Scale [32] and the Movement Assessment Battery for Children-2 [33], which are widely used tools to assess motor development in a municipality setting, but these were not included due to problems with licensing. Moreover, we collected data from a very broad and heterogenous group of children meaning that we could not include condition-specific questionnaires or tools as the main purpose was to compare characteristics and outcome across children receiving physiotherapy in primary health care.

We have previously reported that sex and age distribution as well as cause of referral of the children included in FYSIOPRIM were comparable to those not included [15]. However, only 21.4% of the all children referred to primary health care physiotherapy in the 12-month period was included, reducing the precision of the reported prevalences, and caution should therefore be used when generalising our findings. One reason for this low proportion was difficulties in obtaining parental consent. Initially, participation in the study required written parental consent from both parents, which sometimes was logistically challenging. Halfway through the data collection (after 6 months), we therefore sought ethical approval to obtain written consent from one of the parents, given that the other parent also received written information about the project. Secondly, there were language barriers for non-Norwegian speaking families. Even though consent forms were available in Norwegian and English, parent-report questionnaires were available in Norwegian only. Thus, the included sample reflects children of Norwegian-speaking parents. Thirdly, the PTs had ethical concerns about including families with high burden of care. Even though children in need of habilitation services constitute a substantial part of the physiotherapy services for children and are usually followed for a long period of time, the incidence is low and our sample is therefore likely to contain few of these children as this study included new referrals only.

There was a considerable proportion of children without follow-up data. Assessment of goal attainment and treatment compliance was not relevant for children who only had an examination. However, we did not find baseline differences for most demographic and clinical variables between those with and without follow-up data, but a larger proportion of children referred for orthopaedic concerns and school children had missing follow-up data, and thus we need to be cautious when drawing conclusions about these subgroups.

In order to include a higher number of children receiving physiotherapy services in future studies, we recommend obtaining ethical approval for written consent from one parent only and inclusion of consent forms and questionnaires also in minority languages. To better reflect the whole population of children receiving physiotherapy, including those in need of habilitation services, longer follow-up periods could be considered.

## Conclusions

This longitudinal observational study describes referral patterns, characteristics and treatment outcome of a broad spectrum of children receiving physiotherapy in a primary health care setting in Norway. New referrals included mostly young children, referred from child health care centres due to concerns regarding motor development, asymmetry and orthopaedic conditions. There was partly agreement between causes of referral and the PT’s functional diagnoses, indicating a potential for better collaboration between PTs and public health nurses. The children’s daily activities were little affected by the cause of referral. The majority achieved their main treatment goal and the treatment was carried out as planned. The large variation in the profile of children receiving primary health care physiotherapy shows how primary health care PTs can contribute to fulfil the broad purpose of the primary health care services.

## Supplementary Information

**Additional file 1.** Details of questions from FYSIOPRIM used in this study.

## Data Availability

The datasets generated and/or analysed during the current study are not publicly available due to permission has not been applied for from neither the participants nor the Ethical Committee but might be available from the corresponding author on reasonable request.

## Abbreviations

FYSIOPRIM: Research program for Physiotherapy in Primary Health Care
PT: Physiotherapist
