# Correction to: Profile of children referred to primary health care physiotherapy: a longitudinal observational study in Norway

**DOI:** 10.1186/s12913-021-06099-8

**Published:** 2021-02-05

**Authors:** Kari Anne I. Evensen, Siw Sellæg, Anne-Cath Stræte, Anne E. Hansen, Ingebrigt Meisingset

**Affiliations:** 1 Department of Public Health and Nursing, NTNU, Trondheim, Norway; 2 Department of Clinical and Molecular Medicine, NTNU, Trondheim, Norway; 3 Unit for Physiotherapy Services, Trondheim Municipality, Trondheim, Norway; 4 Department of Physiotherapy, Oslo Metropolitan University, Oslo, Norway

**Correction to: BMC Health Serv Res 21, 16 (2021)**

**https://doi.org/10.1186/s12913-020-05988-8**

Following the publication of the original article [1], it was noted that due to a typesetting error Fig. 2 needs to be updated with a new version, because figure legend is missing in the current version.

**Fig. 2:**
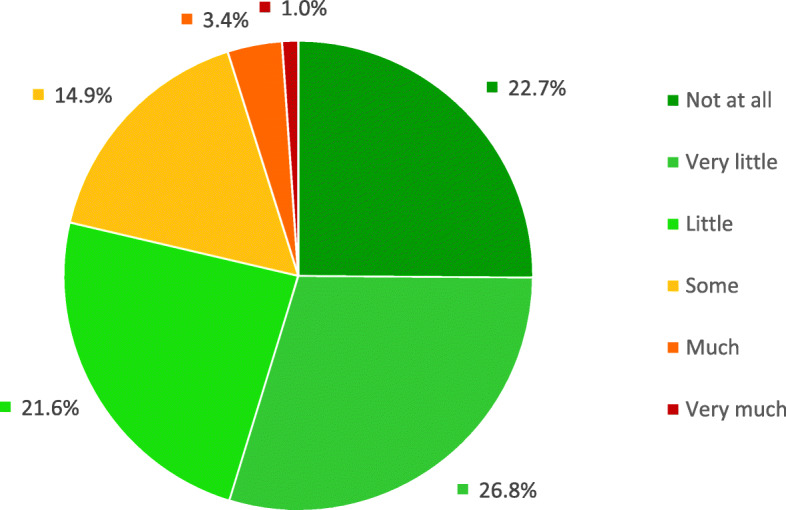
Parent-reported influence of the problem or complaint on the child’s daily activities (*n* = 97)

The updated figure has been included in this correction, and the original article has been corrected.
