# Supplementary material for: Profile of children referred to primary health care physiotherapy: a longitudinal observational study in Norway
Source: BMC Health Serv Res. 2021 Jan 6;21:16. doi: 10.1186/s12913-020-05988-8 (PMC7788700; doi:10.1186/s12913-020-05988-8)
Supplement: Supplementary file 1 — Additional file 1. Details of questions from FYSIOPRIM used in this study. [file 12913_2020_5988_MOESM1_ESM.docx]

**Additional File 1.** Details of questions from FYSIOPRIM used in this study.

| **Name of variable** | **Content description or wording of variable** | **Response option** |
| --- | --- | --- |
| Baseline PT-registration | | |
| Sex | Sex | 1) Girl  2) Boy |
| Age | How old is the child in years? | Age in years |
| Referral source | Referred from: | 1) Child health care centre  2) Hospital  3) Kindergarten  4) School  5) School health care services  6) General practitioner  7) Children’s and family’s services  8) Occupational therapist  9) Proxy/parent  10) Other |
| Cause of referral | Cause of referral: | 1) Motor development  2) Asymmetry (0-1 years)  3) Orthopaedics (gait, foot alignment)  4) Prematurity (0-1 years)  5) Established neurological diagnosis/syndrome  6) Physical activity advice  7) Multidisciplinary assessment  8) Heart or lung disease  9) Other (overweight, juvenile arthritis, cancer, fractures, pain, myalgic encephalomyelitis, referral for assistive devices) |

| PT’s functional diagnosis | What do you consider to be the main problem? | 1. Free text |
| --- | --- | --- |
| Follow-up | Please mark: | 1. 1) Starting physiotherapy 2. 2) Only examination |
| Main treatment goal^a^ | What is the main treatment goal? | 1. Free text |
| Plan for treatment^a^ | What is the most important treatment in order to attain the main treatment goal? | 1. Free text |

| Baseline parent-report | | |
| --- | --- | --- |
| Living situation | Living situation: | 1) Living with both parents  2) Living part time with both parents  3) Living with one parent  4) Other |
|  | Number of siblings with whom the child is living full or part time: | Number of siblings (0-6) |
| Daily arena | The child’s daily arena: | 1. Home 2. Kindergarten 3. School 4. Other |
| Child’s country of birth | Where was the child born? | 1) Norway  2) Europe (EU or EEA)  3) Asia |
| Mother’s country of birth | Where was the mother of the child born? | 1) Norway  2) Europe (EU or EEA)  3) Asia |
| Father’s country of birth | Where was the father of the child born? | 1) Norway  2) Europe (EU or EEA)  3) Asia |
| Parents’ education | Mother’s highest level of education:  Father’s highest level of education: | 1) Primary school or lower  2) High school  3) Up to 4 years of college  4) University/more than 4 years of college or university |
| Prematurity | Was the child born at term (after 37 weeks of gestation)? | 1) Yes  2) No |
| Use of hospital services the last 12 months | Has the child received hospital services for his/her complaints during the last 12 months? | 1) No  2) Yes |
| Pain | If applicable, does the child have pain? | 1) No  2) Yes |
| Influence on daily activities | How much does the problem or complaint affect the child’s daily activities? | 1. 1) Very much   2) Much  3) Some  4) Little  5) Very little  6) Not at all |
| Follow-up PT-registration | | |
| Number of consultations | Number of consultations including this one: | Free text |
| Goal attainment^b^ | To which extent was the main treatment goal achieved? | 1) Achieved  2) Partly achieved  3) Not achieved |
| Treatment compliance^b^ | To which extent was the treatment carried out as planned? | 1) Performed  2) Partly performed  3) Not performed |
| Continued physiotherapy | Is physiotherapy continuing? | 1) Yes  2) No |

EEA = European Economic Area

EU = European Union

FYSIOPRIM = Research program for Physiotherapy in Primary Health Care

PT = Physiotherapist

^a^Identified by the PT and parents together

^b^Assessed by the PT and parents together
